# Evaluation of Serial Procalcitonin Levels for the Optimization of Antibiotic Use in Non-Critically Ill COVID-19 Patients

**DOI:** 10.3390/ph17050624

**Published:** 2024-05-12

**Authors:** Abdulaziz S. Almulhim, Mohammed A. Alabdulwahed, Fatimah F. Aldoughan, Ali M. Aldayyen, Faisal Alghamdi, Rawan Alabdulqader, Norah Alnaim, Dimah Alghannam, Yasmin Aljamaan, Saleh Almutairi, Feras T. Al Mogbel, Ahmad Alamer, Haytham A. Wali

**Affiliations:** 1 Department of Pharmacy Practice, College of Clinical Pharmacy, King Faisal University, Al-Ahsa 31982, Saudi Arabia; asaalmulhim@kfu.edu.sa (A.S.A.); maalabdulwahed@kfu.edu.sa (M.A.A.); faldoughan@kfu.edu.sa (F.F.A.); aaldayyen@kfu.edu.sa (A.M.A.); faisall72@hotmail.com (F.A.); rwepie@gmail.com (R.A.); norah.alnaim@outlook.com (N.A.); i_deemh1100@hotmail.com (D.A.); yasmin_tahir@hotmail.com (Y.A.); 2 Pharmacy Department, King Fahad Military Medical Complex, Dhahran 31932, Saudi Arabia; salehm@kfmmc.med.sa (S.A.); ffeeraass@gmail.com (F.T.A.M.); 3 Department of Clinical Pharmacy, Prince Sattam Bin Abdulaziz University, Alkharj 11942, Saudi Arabia; aa.alamer@psau.edu.sa

**Keywords:** procalcitonin, antibiotic use, COVID-19, pneumonia, antimicrobial stewardship

## Abstract

Background: Procalcitonin (PCT) has been used as a biomarker to guide antibiotic therapy in various patient populations. However, its role in optimizing antibiotic use in COVID-19 patients has not been well studied to date. Thus, we aimed to evaluate the use of serial PCT monitoring as an antimicrobial stewardship tool for COVID-19 patients. Methods: This retrospective study included 240 COVID-19 patients who were admitted to a tertiary medical institution in Saudi Arabia between January 2020 and February 2022. Patients who received empiric antibiotic therapy for community-acquired pneumonia (CAP) and had serial procalcitonin levels were included. The patients were divided into two groups: the normal procalcitonin arm (PCT level < 0.5 ng/mL) and the elevated PCT arm (PCT level > 0.5 ng/mL). The primary and secondary outcomes were the effect of PCT monitoring on the duration of antibiotic exposure and the length of hospital stay, respectively. To measure the accuracy of PCT, the receiver-operating characteristic area under the curve (ROC-AUC) was determined. Results: Among the included patients, 142 were in the normal procalcitonin arm (median PCT, 0.12 ng/mL), and 78 were in the elevated PCT arm (median PCT, 4.04 ng/mL). The baseline characteristics were similar between the two arms, except for the higher prevalence of kidney disease in the elevated PCT arm. There was no statistically significant difference in the duration of antibiotic exposure between the normal and elevated PCT arms (median duration: 7 days in both arms). However, the length of hospital stay was significantly shorter in the normal PCT arm (median stay, 9 days) than in the elevated PCT arm (median stay, 13 days; *p* = 0.028). The ROC-AUC value was 0.54 (95% CI: 0.503–0.595). Conclusions: Serial PCT monitoring did not lead to a reduction in the duration of antibiotic exposure in COVID-19 patients. However, it was associated with a shorter hospital stay. These findings suggest that PCT monitoring may be useful for optimizing antibiotic use and improving outcomes in COVID-19 patients. While PCT-guided algorithms have the potential to enable antibiotic stewardship, their role in the context of COVID-19 treatment requires further investigation.

## 1. Introduction

Although antibiotics have revolutionized medicine, their use has drastically altered bacterial resistance patterns. In Saudi Arabia, antimicrobial resistance is a growing threat to public health and is the third leading cause of death [1]. This change in the resistance of strains has encouraged the scientific community to find alternative pathways for the optimization of antibiotic use. Biomarkers have been introduced as tools for diagnosing and predicting the time course of specific illnesses and therefore the length of antibiotic treatment needed [2]. Due to its specificity, procalcitonin (PCT) has gained popularity in the field of infectious diseases. PCT has primarily been used to guide antibiotic therapy in selected patient populations [3]. PCT is a precursor of calcitonin and consists of 116 amino acids. Its level is usually elevated in response to inflammation; however, concentrations above 1 ng/mL indicate severe bacterial infection [4]. Multiple trials have used procalcitonin to guide the discontinuation of antibiotics without increasing mortality, particularly in patients with sepsis or septic shock [5].

Coronavirus disease 2019 (COVID-19), caused by SARS-CoV-2, primarily affects the lungs and immune system, with symptoms including fever, cough, and dyspnea [6]. Severe cases are characterized by a combination of pulmonary macrophage activation, complement-mediated endothelialitis, and a procoagulant state [7]. Long COVID-19, denoting post-acute sequelae, is associated with viral persistence, hypercoagulopathy, immune dysregulation, and hyperinflammation [8]. The virus can also affect the nervous system, leading to a range of mild-to-severe neurological symptoms, including cerebrovascular disease [9].

Bacterial co-infections are common among critically ill patients. These may complicate the clinical picture and increase mortality rates [10,11]. Additionally, robust data have indicated an association between early and proper antibiotic therapy and survival in patients with sepsis [12]. However, data from a systematic review and meta-analysis of 22 hospital-based studies comprising 76,176 COVID-19 patients indicated that the prevalence of bacterial co-infection in COVID-19 is low (5.62%; 95% CI: 2.26–10.31); however, the prevalence of antibiotic prescription is 61.77% (95% CI: 50.95–70.90) [13], which can be a reason for COVID-19 antimicrobial stewardship initiatives to reduce antimicrobial resistance.

Given the sensitivity of PCT to bacterial toxins, high concentrations of this compound are associated with severe inflammatory responses and bacterial infections [3,14,15]. Delévaux et al. evaluated the possible discriminative role of PCT as a biomarker in differentiating bacterial infections from other inflammatory processes, where PCT > 0.5 ng/mL was used as the cut-off level for bacterial infection (sensitivity, 65%; specificity, 96%). PCT levels only increase significantly during bacterial infections [16]. Several studies on sepsis, septic shock, and pneumonia patients have proposed and validated PCT-guided algorithms with different cut-off levels [17,18,19,20,21,22,23]. In non-ICU COVID-19 patients, PCT levels can help to identify those at risk of bacterial co-infections, with a cut-off value of 0.25 ng/mL showing a poor positive predictive value but a high negative predictive value. However, the use of antibiotics to treat non-ICU COVID-19 patients with elevated PCT levels did not improve clinical outcomes [24]. Moreover, few studies have examined PCT as a stewardship tool to decrease the use and duration of broad-spectrum antibiotics in COVID-19 patients [25,26].

Further research is needed to fully understand the role of PCT in non-ICU COVID-19 patients. Therefore, this study examined procalcitonin as an antimicrobial stewardship tool for the optimization of antibiotic use in these patients.

## 2. Results

### 2.1. Baseline Characteristics

**Figure 1:**
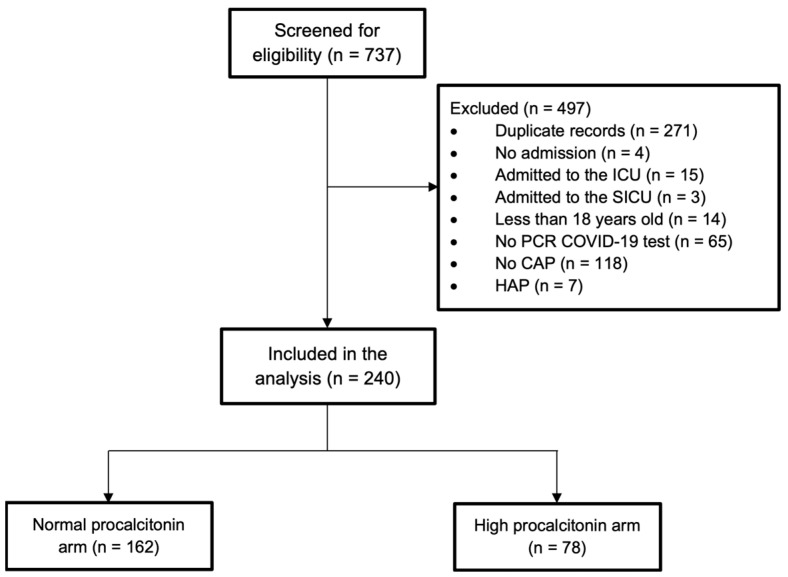
Flowchart of the patients included in this study. ICU: intensive care unit; SICU: surgical intensive care unit; PCR: polymerase chain reaction; COVID-19: Coronavirus disease 2019; CAP: community-acquired pneumonia; HAP: hospital-acquired pneumonia.

A total of 737 electronic medical records were screened for eligibility, and based on this, 497 patients were excluded. The reasons for exclusion are shown in Figure 1. After removing duplicate records, 240 patients were included in the analysis and divided into two arms based on their PCT levels (normal vs. high). The first arm included 162 patients (normal procalcitonin, 0.12 ng/mL) and the second included 78 patients (high procalcitonin, 4.04 ng/mL). The baseline characteristics were similar between the two arms, except that there were significantly more patients with kidney disease in the high-PCT arm (38.5%) than in the normal-PCT arm (16%; *p* < 0.001). Serum creatinine and blood urea nitrogen (BUN) concentrations were elevated in the high-PCT group. The median white blood cell (WBC) count was higher in the high-PCT arm than in the normal-PCT arm (7.8 vs. 5.6 × 10^9^/L; *p* < 0.001). Moreover, the complete blood count (CBC) differential showed statistically significant differences in terms of the lymphocyte and neutrophil percentages between the two arms (*p* < 0.001). The baseline patient characteristics are presented in Table 1.

### 2.2. Study Outcomes

**Figure 2:**
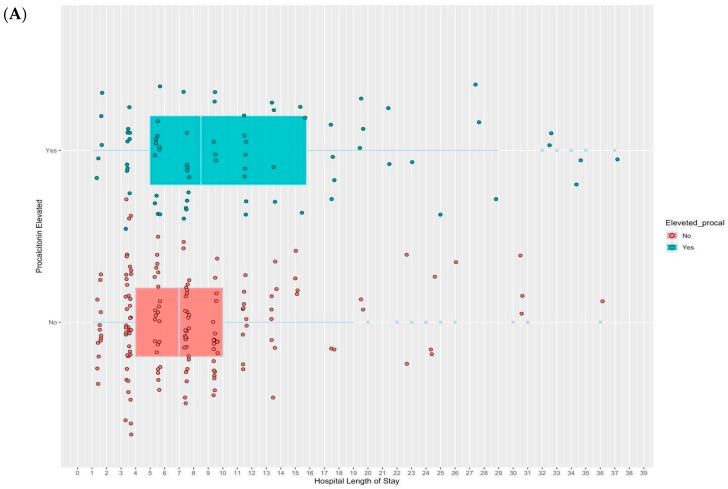

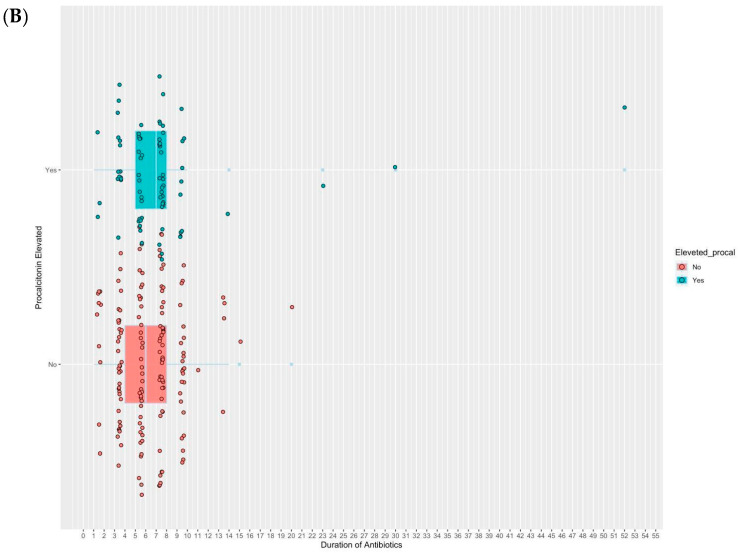
Raw data for the duration of hospital stay (**A**) and antibiotic exposure (**B**). Each point represents an individual patient.

**Figure 3:**
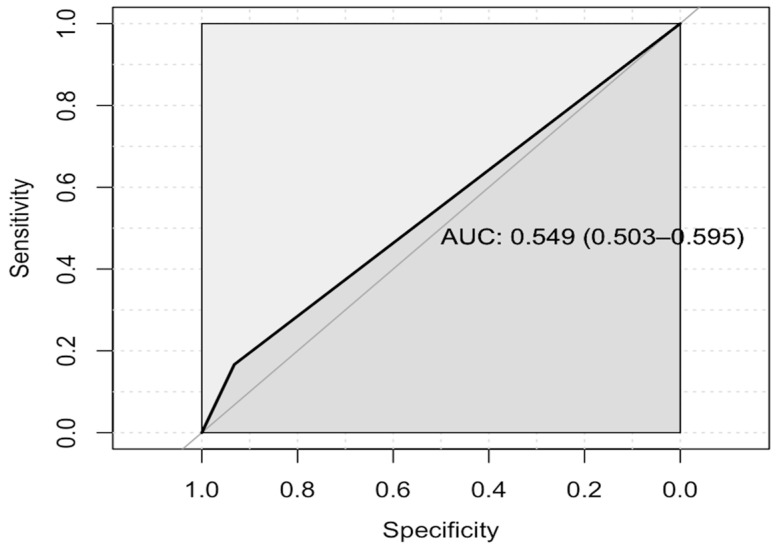
Receiver-operator characteristic area under the curve (ROC-AUC) for procalcitonin (PCT) levels as a discriminatory measure of positive blood or respiratory cultures.

There was no statistical difference in the antibiotics course employed between the normal-PCT arm and the elevated procalcitonin arm (7 days vs. 8.5 days; estimated median difference of 0.21, OR = 1.2, 95% credible interval (CI): 0.8–1.9). Nevertheless, a statistically significant difference was found between the two arms regarding the length of hospital stay, with a median of 6 vs. 7 days for the normal-PCT arm vs. the high-PCT arm (estimated median difference 0.73, OR = 2, 95% CI: 1.3–3.4); see Table 2. Raw data for the duration of hospital stay and antibiotic exposure are shown in Figure 2. The ROC discriminatory power of PCT for positive blood or respiratory cultures was 0.54 (95% CI: 0.503–0.595), as shown in Figure 3.

## 3. Discussion

This retrospective observational study evaluated the value of PCT monitoring as an antimicrobial stewardship tool for COVID-19 patients. Although no reduction in the duration of antibiotic exposure was observed, the length of hospital stay was significantly reduced in the normal-PCT arm.

Given the heterogeneous nature of infections in critically ill patients, differentiating between bacterial and non-bacterial etiologies is complex [27]. This commonly leads to the unnecessary use of antibiotics, which is estimated to be between 30and 40% in hospitalized settings [28]. Regarding irrational antibiotic use, the COVID-19 pandemic was not an exception. Several studies have evaluated antibiotic use during the pandemic [29,30]. Vâţă et al. conducted a retrospective study to assess antibiotic use practices in critically ill patients. Unsurprisingly, all admitted patients (N = 184) received antibiotics; however, microbiological confirmation of the disease was only conducted in 18 patients (9.8%). Concerning PCT, only 38% had elevated levels, although bacterial co-infection was assessed using other clinical or biological criteria [31]. Choi et al. retrospectively reviewed Korea’s National Health Insurance Service (NHIS) database to evaluate antibiotic use during the pandemic. Among the critical COVID-19 cases (N = 2484), 73.8% received antibiotics. Due to the nature of the claimed data, neither bacterial co-infection nor PCT levels were assessed [32]; however, caution should be exercised when interpreting the results due to the lack of serial PCT monitoring and bacterial culture confirmation.

Before the pandemic declaration, PCT was utilized as an antimicrobial stewardship tool to differentiate between bacterial and viral infections and guide antibiotic use [33,34,35]. In patients with sepsis, it showed excellent diagnostic accuracy, with an area under the receiver-operating characteristic curve (ROC curve) of 0.85 (95% CI: 0.81–0.88) [36]. Therefore, serial PCT monitoring has been recommended as a diagnostic and monitoring tool for patients with possible sepsis [37]. Bouadma et al. conducted a randomized controlled study to evaluate the effectiveness of a PCT-guided strategy on mortality and duration of antibiotic use. Although the study population consisted mainly of patients with sepsis, respiratory infections were the most common source of disease (73%). Using a cut-off value of 0.5 ng/mL, antibiotic discontinuation was effective for reducing the antibiotic course (14.3 days vs. 11.6 days, 95% CI: 1.4 to 4.1, *p* < 0.0001) with no unfavorable outcomes [18]. Similarly, Jong et al. conducted a randomized controlled study to assess the safety and efficacy of PCT as an antibiotic-guided tool [36]. Pulmonary infections were the most common source of infection (65%). Again, PCT utilization was associated with a shorter antibiotic duration in PCT-guided groups compared to standard care groups (5 days vs. 7 days, 1.22, 95% CI: 0.65–1.78). 

Since the declaration of the COVID-19 pandemic, many blood markers have been used to characterize the degree of severity of the disease and as diagnostic tools [27,37,38,39]. C-reactive protein (CRP) and PCT levels are of great interest in this regard. Hu et al. conducted a retrospective study to evaluate the association between PCT and disease severity. PCT levels were highly correlated (mean 0.44 ± 0.55 ng/mL) with disease severity. It is worth noting that the co-infection rate in critically ill patients was 50% [27].

Although PCT is highly sensitive as a diagnostic tool, it has low specificity, as it can be elevated in non-bacterial infections and non-infectious etiologies [40]. In addition to its diagnostic value, the PCT level has been found to predict the severity of COVID-19 infection. Minh et al. investigated the relationship between the COVID-19 infection severity and the PCT level. Compared to non-severe COVID-19 infections, severe COVID-19 infections were associated with elevated PCT levels (0.1 ng/mL vs. 0.47 ng/mL, *p* < 0.001). However, after adjusting for bacterial co-infection and other factors, the elevation of the PCT level was associated with severe COVID-19 infection with an OR of 2.11 (95% CI: 1.36–3.61), again signaling its low specificity in the setting of bacterial and COVID-19 infections [37]. Carbonell et al. conducted another retrospective study to investigate the predictive value of PCT for bacterial co-infection in COVID-19 patients. PCT performed poorly, with an AUC of 0.56 (95% CI: 0.53–0.59). Using a cut-off value of <0.3 ng/mL, PCT was shown to have a negative predictive value of 91.1% (95% CI: 90.0–92.2) [26]. Alnimr et al. examined the predictive role of PCT in critically ill COVID-19 and bacteria-co-infected patients. Almost all non-survivors (67 out of 68) had bacterial and fungal co-infections, with a median PCT of 1.6 ng/mL (±4.7) compared to 0.2 ng/mL (±4.2). Nevertheless, bacterial co-infection was not reported in the survivors [41]. Our study’s AUC of 0.55 confirmed the low discriminatory power of PCT in patients with COVID-19. The results showed that the median PCT level in the elevated PCT arm was 4.04, with only 10.5% and 10% of patients having positive blood and respiratory cultures, respectively.

The present study had several limitations. First, our study was conducted during the early period of the pandemic, when many parameters were missing. For instance, D-dimer and C-reactive protein levels were not collected, given that their roles as inflammatory and severity indicators in COVID-19 were unknown during the early stages of the pandemic. The lack of significance in antibiotic duration could be explained by the absence of treatment guideline algorithms (i.e., PCT-guided antibiotic therapy). Rather than being a specific test for bacterial infections, the low specificity of PCT could explain its elevation in the elevated PCT level arm. Second, as this study was a retrospective chart review and not a randomized clinical trial, we could not control the hospitals’ practices in terms of prescribing antibiotics. The PCT level is mainly used to aid in the discontinuation of antibiotics, rather than the initiation of their use. Nevertheless, there were still deviations from proper practice, which were reflected in the duration of antibiotic use in the study groups. Third, data on oxygen saturation on admission were difficult to collect from the hospital’s electronic medical records; therefore, they were not included in the collected data. Fourth, it is worth considering that using multiplex PCR technology can be more advantageous than measuring PCT levels in addition to conventional microbiological identification methods for distinguishing between viral and bacterial infections. However, using such technology is subject to its availability at the hospital and one’s ability to afford its cost. In our study, the hospital did not have this technology, so a direct comparison between the two approaches could not be provided.

Future research should focus on several key aspects. First, additional studies are needed to validate the findings of this study and further investigate the utility of serial PCT monitoring in different subsets of COVID-19 patients, including those with varying levels of disease severity and comorbidities. Understanding how PCT levels correlate with bacterial coinfections and their impact on clinical outcomes will help to refine PCT-guided algorithms for antibiotic use in this specific patient population. Second, prospective studies should evaluate the cost-effectiveness of PCT monitoring for antimicrobial stewardship programs in the context of COVID-19. Assessing the economic implications, such as potential reductions in antibiotic usage and hospitalization costs, will provide valuable insights for healthcare decision makers. Additionally, future research should explore the integration of PCT monitoring with other biomarkers and clinical parameters, in order to develop comprehensive and personalized approaches for antibiotic management in COVID-19 patients. Finally, as the understanding of COVID-19 evolves, it is crucial to adapt and update the guidelines and recommendations regarding PCT monitoring and its role in optimizing antibiotic therapy. Ongoing research and collaboration among multidisciplinary teams will contribute to refining strategies for antimicrobial stewardship and improving patient outcomes in the context of COVID-19.

## 4. Materials and Methods

### 4.1. Study Design and Setting

This comparative single-center retrospective chart review evaluated the effect of serial PCT monitoring on antibiotic discontinuation in patients with COVID-19. It was conducted at the King Fahd Military Medical Complex (KFMMC) in Dhahran, Saudi Arabia, a 300-bed tertiary medical institution at the Armed Forces Medical Services in Saudi Arabia.

### 4.2. Study Procedures and Participants

All COVID-19 patients admitted between January 2020 and February 2022 were identified. Electronic medical records (EMRs) were screened for eligibility between January 2020 and February 2022. Patients were included if they were hospitalized, aged 18 years or older, had a confirmed COVID-19 infection (via real-time polymerase chain reaction; RT-PCR), received empiric antibiotic therapy upon admission, and had a serial PCT order. We excluded patients younger than 18 years or those with one of the following: an infection on admission other than COVID-19 or community-acquired pneumonia, antibiotics administered in the community before admission, suppressive antibiotic therapy for chronic infections, documented infections requiring prolonged antibiotic therapy (e.g., endocarditis or osteomyelitis), or severe immunosuppression.

The study population included two arms of patients using convenience sampling [42]. Patients with COVID-19 who had received empiric antibiotic therapy for community-acquired pneumonia (CAP) and had normal serial PCT levels (defined as procalcitonin level < 0.5 ng/mL) were referred to as the normal procalcitonin arm. The second arm (elevated PCT arm) included patients diagnosed with COVID-19 who had received empiric antibiotic therapy for CAP and had high serial PCT levels (PCT level > 0.5 ng/mL).

The hospital used ALINITY i B·R·A·H·M·S PCT (Thermo Fisher Scientific Inc., Waltham, MA, USA), a chemiluminescent microparticle immunoassay (CMIA), for the quantitative determination of PCT. For bacterial identification and antimicrobial susceptibility testing (ID/AST), the hospital used the DxM MicroScan WalkAway ID/AST System (Beckman Coulter, Inc., Brea, CA, USA).

### 4.3. Study Outcomes

The primary outcome of this study was to assess the impact of PCT-level monitoring on the duration of antibiotic exposure in COVID-19 patients. The secondary outcome was to determine the effect of PCT-level monitoring on the length of hospital stay.

### 4.4. Ethical Consideration

The study protocol was approved by the Armed Forces Hospitals Eastern Region Institutional Review Board (IRB) under reference number AFHER-IRB-2022-015 and King Faisal University IRB under reference number KFU-REC-2022-OCT-ETHICS234. As the study was retrospective, informed consent was not deemed necessary. Additionally, no patient information was collected beyond the requirements for data analysis.

### 4.5. Sample Size Calculation

The sample size was calculated using the Hmisc package with the posamsize function in R (v4.1.2; Vienna, Austria) [43]. Based on an odds ratio of 2, the total sample size required to detect statistical differences between the two groups was estimated to be 198. The alpha level was 0.05 and the power was 80%. The design was shown to be highly efficient, with an efficiency of 0.99 when compared to the continuous response.

### 4.6. Statistical Analysis

Categorical variables are presented as frequencies and percentages and were compared using either the Chi-square or Fisher’s exact test. Parametric continuous variables are presented as means and standard deviations (SD), while non-parametric continuous variables are presented as medians and interquartile ranges (IQR). Continuous variables were compared using Student’s *t*-test and the Mann–Whitney U-test for parametric and non-parametric data, respectively.

The outcomes of interest (i.e., duration of antibiotic exposure or length of hospital stay measured in days) were treated as the ordinal variables. Therefore, we applied an ordinal regression model to explain the response variables. The advantage of using such a model is that no distributional assumption exists for Y (response variable), given an explanatory variable X [44]. In addition, durations are usually heavily tailed and skewed. Therefore, the normality assumptions are rarely met in this setting. The Bayesian framework allowed us to present the point of estimates as odds ratios with 95% credible intervals. This analysis was performed using the BRMS package in R [45]. The causal assumptions were drawn using directed acyclic graphs (DAGs); there was no biasing pathway between procalcitonin level and duration of antibiotics that would necessitate the inclusion of other variables in the model (Figure S1). Data visualization was performed using the ggplot2 package in R [46]. The discriminatory measure of procalcitonin levels in positive blood or respiratory cultures was assessed using the receiver operating characteristic area under the curve (ROC-AUC). All analyses were performed using R Core Team software (R Foundation for Statistical Computing, Version (v4.1.2; Vienna, Austria)).

## 5. Conclusions

In conclusion, this study investigated the role of serial PCT monitoring as an antimicrobial stewardship tool for patients with COVID-19. While our findings did not demonstrate a reduction in the duration of antibiotic exposure with the use of this biomarker, they did reveal a significantly shorter length of hospital stay in patients with normal PCT levels. These results suggest that PCT monitoring may enable the optimization of antibiotic use, thus improving outcomes in non-critically ill COVID-19 patients. However, further research is needed to explore the potential of PCT-guided algorithms in antimicrobial stewardship initiatives specifically tailored to COVID-19 patients. Through refining our understanding of the role of PCT, we can enhance the rational use of antibiotics, minimize the development of antimicrobial resistance, and improve patient care in the context of this global pandemic.

## Figures and Tables

**Table 1:** Baseline characteristics of the study participants.

Characteristic	Normal Procalcitonin (*n* = 162)	Elevated Procalcitonin (*n* = 78)	*p*-Value
Age, years, median (IQR)	57.5 (46.7–70.0)	64.0 (50.3–72.8)	0.269
Male, n (%)	92 (56.8)	47 (60.3)	0.711
Weight, kg, median (IQR)	85.5 (72.5–96.0)	76.0 (64.0–93.8)	0.069
SBP, mmHg, mean (SD)	133.0 (117.8–146.0)	128.5 (117.0–140.5)	0.133
DBP, mmHg, mean (SD)	71.54 (63.75–79.25)	70.27 (70.0–77.5)	0.465
BUN, mg/dL, median (IQR)	4.8 (3.4–7.2)	7.6 (4.8–13.3)	<0.001
SCr, mmol/L, median (IQR)	83.0 (67.0–102.0)	105.5 (76.7–206.5)	<0.001
Lactate, mmol/L, median (IQR)	1.4 (1.0–1.8)	1.5 (1.0–1.9)	0.294
WBC, ×10^9^/L, median (IQR)	5.6 (4.1–7.3)	7.8 (5.1–11.4)	<0.001
Neutrophils in %, median (IQR)	67.4 (57.7–77.0)	76.0 (64.6–83.9)	<0.001
Lymphocyte in %, median (IQR)	23.1 (15.50–30.2)	16.9 (8.1–22.2)	<0.001
CURB-65, median (IQR)	1.0 (0–1)	1.0 (0.0–2.0)	0.009
Comorbidities			
Hypertension, n (%)	79 (48.8)	46 (59.0)	0.179
Diabetes, n (%)	69 (42.6)	43 (55.1)	0.092
Heart disease, n (%)	34 (21.0)	23 (29.5)	0.198
Kidney disease, n (%)	26 (16.0)	30 (38.5)	<0.001
Liver disease, n (%)	10 (6.2)	1 (1.3)	0.171
Lung disease, n (%)	18 (11.1)	6 (7.7)	0.550
Immunocompromised status, n (%)	12 (7.4)	8 (10.3)	0.618
Neoplastic disease, n (%)	1 (0.6)	4 (5.1)	0.070
Cerebrovascular disease, n (%)	15 (9.3)	8 (10.3)	0.991
Microbiological Data *			
Positive respiratory culture, n (%)	3 (3.4)	5 (6.4)	0.225
Positive blood culture, n (%)	9 (6.6)	8 (10.3)	0.459
Positive respiratory or blood culture, n (%)	11 (6.8)	13 (16.7)	0.031
Procalcitonin level, ng/mL, median (IQR)	0.12 (0.05–0.13)	4.04 (0.35–2.27)	<0.001
Antibiotics on Admission			
Azithromycin, n (%)	140 (87.5)	61 (78.2)	0.096
Ceftriaxone, n (%)	133 (83.1)	61 (78.2)	0.459
Other, n (%) ^†^	57 (33.3)	31 (40.3)	0.362

* Identified bacteria included the following: *Escherichia coli*, *Klebsiella pneumoniae*, Methicillin-resistant *Staphylococcus aureus*, *Pseudomonas aeruginosa*, *Staphylococcus capitis*, *Staphylococcus epidermidis*, *Streptococcus agalactiae*, and *Streptococcus mitis*. ^†^ Other antibiotics included: cefepime, ceftazidime, cefuroxime, ciprofloxacin, ertapenem, linezolid, meropenem, metronidazole, piperacillin-tazobactam, and vancomycin. IQR: interquartile range; SD: standard deviation; SBP: systolic blood pressure; DBP: diastolic blood pressure; BUN: blood urea nitrogen; SCr: serum creatinine; WBC: white blood cells; CURB-65: confusion, uremia, respiratory rate.

**Table 2:** Primary and secondary endpoints results (Odds Ratio, 95% credible intervals).

Outcome	Normal Procalcitonin *n* = 162	Elevated Procalcitonin *n* = 78	Absolute Median Difference (95% Confidence Interval)	Estimated Median Difference (95% Credible Interval)	Odds Ratio (95% Credible Interval)
LOS, median (IQR)	6.0 (4.0–8.0)	7.0 (5.0–8.0)	1.0 (−0.04 to not estimated)	0.73 (0.25–1.21)	2.0 (1.3–3.4)
Antibiotics duration, median (IQR)	7.0 (4.0–10.0)	8.5 (5.0–15.8)	1.5 (−3.0 to 1.5)	0.21 (−0.25 to 0.68)	1.2 (0.8–1.9)

Bayesian proportional odds model. The estimated median differences were derived by fitting these models. LOS: length of stay; IQR: interquartile range. The absolute confidence intervals were estimated using a quantile regression model.

## Data Availability

The data sets generated or analyzed during the current study are available from the corresponding author upon reasonable request.

## Supplementary Materials

The following supporting information can be downloaded at: https://www.mdpi.com/article/10.3390/ph17050624/s1, Figure S1: Directed acyclic graph showing the underlying causal assumption between procalcitonin level and antibiotic duration.
