# Supplementary material for: Evaluation of Serial Procalcitonin Levels for the Optimization of Antibiotic Use in Non-Critically Ill COVID-19 Patients
Source: Pharmaceuticals (Basel). 2024 May 12;17(5):624. doi: 10.3390/ph17050624 (PMC11124043; doi:10.3390/ph17050624)
Supplement: Supplementary file 1 [file pharmaceuticals-17-00624-s001.zip › pharmaceuticals-2921490-supplementary.pdf]

Supplementary Materials

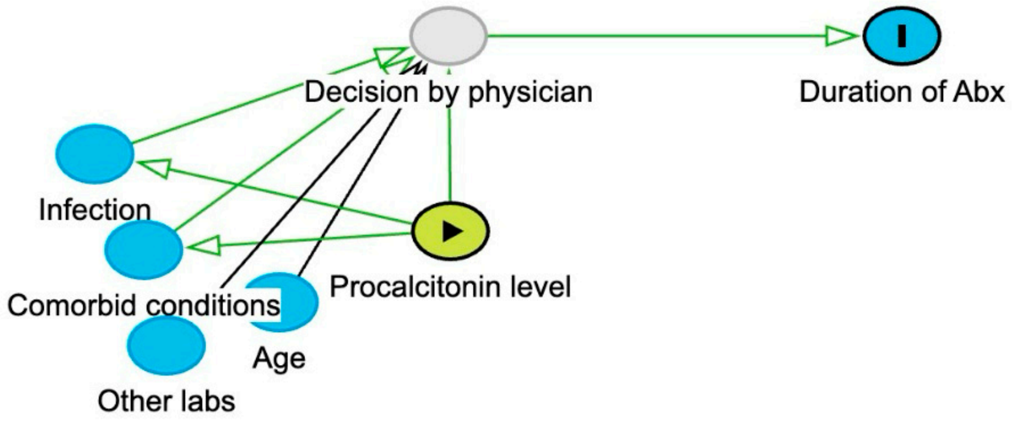

Figure S1. Directed acyclic graph for the underlying causal assumption between procalcitonin level and duration of antibiotics. The decision by the provider is an unobserved variable, and there was no biasing pathway to estimate the causal pathway between procalcitonin levels and the duration of antibiotics.
